# Effect of oral Utrogestan in comparison with Cetrotide on preventing luteinizing hormone surge in IVF cycles: A randomized controlled trial

**DOI:** 10.18502/ijrm.v18i1.6197

**Published:** 2020-01-27

**Authors:** Alieh Ghasemzadeh, Masumeh Dopour Faliz, Laya Farzadi, Nazli Navali, Behzad Bahramzadeh, Arash Fadavi, Parvin Hakimi, Sepideh Tehrani-ghadim, Sedigheh Abdollahi fard, Kobra Hamdi

**Affiliations:** Women's Reproductive Health Research Center, Tabriz University of Medical Sciences, Tabriz, Iran.

**Keywords:** In vitro fertilization, Premature luteinization, Utrogestan.

## Abstract

**Background:**

Oral progesterone is recommended as an alternative to gonadotropin-releasing hormone (GnRH) agonists and antagonists to prevent luteinizing hormone (LH) surge in assisted reproductive technology (ART) cycles. However, there are little data regarding its use.

**Objective:**

We aimed to compare the effect of oral Utrogestan and Cetrotide (a GnRH antagonist) on preventing LH surge in ART cycles.

**Materials and Methods:**

In this randomized clinical trial, 100 infertile women undergoing ART who received recombinant follicle-stimulating hormone (FSH) at 150-225 IU/day were randomly assigned to receive either Utrogestan 100 mg twice a day (case group) or GnRH antagonist protocol (control group) from cycle day 3 until the trigger day. Triggering was performed with 10,000 IU hCG) when there were at least three mature follicles. Viable embryos were cryopreserved for transfer in the next cycle for both groups. The number of oocytes retrieved and transferred embryos were compared between groups.

**Results:**

The case group had significantly higher progesterone levels on triggering day, more follicles of >14 mm with higher maturity, and more oocytes retrieved with a higher rate of embryos transferred. A small increase in the pregnancy rate was observed in the case group, with no significant between-group differences. The most important result was the lack of premature LH surge in either group upon serum LH assessment on the triggering day.

**Conclusion:**

Utrogestan is an alternative treatment that could reduce the LH surge rate and increase the ART outcomes including the number of oocytes retrieved and transferred embryos compared with GnRH agonists and antagonists

## 1. Introduction

Different assisted reproductive technology (ART) methods have been used for the treatment of infertility (1, 2). The success rates of ART have increased with the use of controlled ovarian hyperstimulation which increases the number of oocytes retrieved. However, premature luteinizing hormone (LH) surge is a great burden that reduces the treatment efficacy. Gonadotropin-releasing hormone (GnRH) agonists and antagonists significantly reduced LH surge in in vitro fertilization (IVF) cycles (3-5). Zhu and colleagues reported that progesterone soft capsules (Utrogestan) could prevent premature LH surge in IVF cycles (6, 7). This treatment along with hMG protocol had higher retrieved viable embryo rate per retrieved oocyte than short protocol. However, few studies have evaluated the effects of progesterone, including Utrogestan, on preventing LH surge and improving ART outcomes.

Progesterone is the primary modulator of GnRH pulse frequency slowing in women. The increased progesterone levels in luteal phase causes slowing of the LH and GnRH pulse frequency, which also happens in follicular phase after administering progesterone. The proper level for progesterone causing LH pulse frequency suppress in productive women is uncertain (8).

In this study, we aimed to evaluate the effects of oral Utrogestan in comparison with Cetrotide on preventing LH surge in ART cycles.

## 2. Materials and Methods

In this randomized single-blinded controlled clinical trial, 100 infertile women undergoing IVF at Al-Zahra Hospital, Tabriz, Iran, were included. Infertile women aged 20-40 years with an antral follicle count of >4 on day 3 of the cycle and a baseline FSH of <15 IU/L were included. If there was any contraindication for ovarian stimulation or evidence of ovarian failure, endometriosis grades III and IV or severe male factor as the infertility cause the patients were excluded from the study. Cycles using donor oocytes or cryopreserved embryos were excluded from this study. We also excluded women with known intrauterine anomalies, previous abortion or curettage, operative hysteroscopy, polypectomy, myomectomy or septum resection.

**Figure 1:**
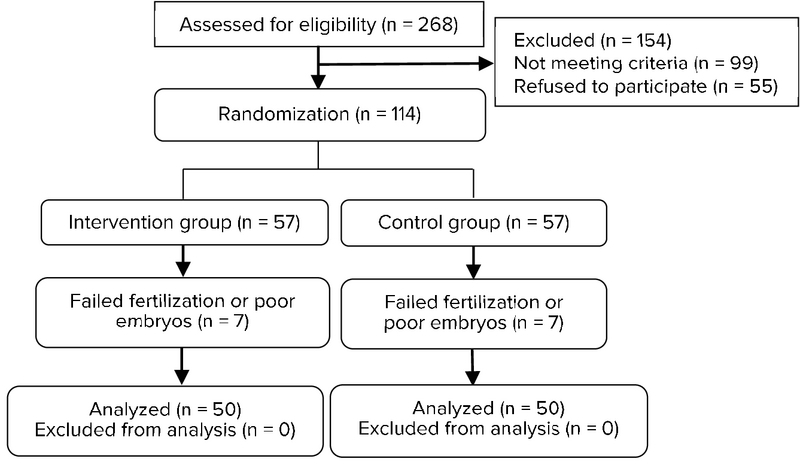
Study Flow diagram.

Using G*Power software 9.3.2, the sample size was calculated 46 per condition, considering an effect size of d ≥ 0.70 as statistically significant in a two-tailed test with α = 0.05 and power of 0.8. To prevent patient loss during the study and maintain the study power, we recruited 57 patients for each group. Patients were enrolled in the study via block randomization (Figure 1). After being selected, women were randomly allocated to either the case and control group. All patients in both groups received recombinant FSH (GonalF, Merck serono) at 150-225 IU per day since day 2/3 of menstruation until the trigger day. The case group received Utrogestan 100 mg (Esins health care) twice a day from day 3 of menstruation, whereas the control group was treated with GnRH antagonist protocol. Transvaginal sonography was performed and after detecting mature follicle (≥ 14 mm), Cetrotide 0.25 mg (cetrorelix acetate, Merck Serono Ltd) was subcutaneously injected every day until HCG injection day in the control group. We prescribed 10,000 unit hCG (Pregnyl, Organon, Netherland) when there were at least three mature follicles. Oocyte retrieval planned for 36 hr after hCG triggering, and fertilization of retrieved oocytes was performed via IVF or intracytoplasmic sperm injection. In both groups, viable embryos were cryopreserved for later, which was performed in the next cycle. A maximum of three embryos was implanted into each patient. After implantation, progesterone (in oil; 50 mg) was administered Intramuscular twice daily to support luteal function. Pregnancy was confirmed by positive serum β-HCG 14 days after embryo transfer. The determination of gestational sac(s) through ultrasonography exam was a sign of clinical pregnancy. The progesterone and LH levels at cycle day 3 and trigger day were measured.

### Ethical consideration

The ethics committee of the Tabriz University of Medical Sciences approved this study (code: IR.TBZMED.REC.1396.363), and all patients provided informed consent.

### Statistical analysis

The statistical analysis was performed using SPSS 20 (Statistical Package for the Social Sciences, SPSS Inc., Chicago, IL, U.S.A.). Results were presented as mean and standard deviation (SD) or frequency and percentage. An independent *t*-test and chi-squared test were used to compare the results between groups. P-values of <0.05 were considered significant.

## 3. Results

As shown in Figure 1, 268 eligible women were evaluated, and 114 women were included in the study. Seven women from each group were excluded owing to failed fertilization or poor embryos. Finally, 100 women underwent IVF in the case (n = 50) and control (n = 50) groups. Table I presents their demographic findings, with no difference between the groups. The most common cause of primary infertility was the tubal factor. The clinical manifestations and findings following treatment are summarized in Table II. The case group had significantly higher LH at day 3 of the cycle, progesterone at the HCG day, and more follicles of >14 mm with higher maturity. In addition, more oocytes were retrieved in this group, and a higher rate of embryos were transferred. Positive pregnancy tests were higher in the case group, with no significant difference. LH levels significantly decreased in both the case (p < 0.001) and control groups (p = 0.001) at the HCG injection day compared with baseline. There was no premature LH surge in either group.

**Table 1:** Baseline findings between groups


	<statement> <title>Case </title> </statement>	**Control**	**P-value**
Age (yr)*		30.22 ± 6.20	32.40 ± 6.57	0.09
	Primary	39 (78%)	11 (22%)	
Infertility**	Secondary	33 (66%)	17 (34%)	0.18
		Tubal factor	24 (48%)		20 (40%)	
	Male factor	17 (34%)	24 (48%)	
Causes of infertility	Unexplained	9 (18%)	6 (12%)	0.34
	Basal FSH	5.80 ± 2.16	4.96 ± 1.70		0.03
	Previous IVF	3 (6%)	7 (14%)		0.18
1-2 times		2	5	
>3 times		1	2	—-
Data presented as Mean ± SD or frequency (percent), * Independent samples t-test; ** Chi-square test				
FSH: Follicle-stimulating hormone; IVF: In vitro fertilization				

**Table 2:** Clinical manifestations and findings following treatment


	<statement> <title>Case </title> </statement>	**Control**	**P-value**
	Day 3 of cycle	7.53 ± 4.85	4.74 ± 2.67	0.001
LH*	HCG day	3.88 ± 2.54	2.74 ± 3.23	0.052
		Day 3 of cycle	7.75 ± 1.58	0.65 ± 0.10	0.54
Progesterone	HCG day	11.14 ± 1.69	0.90 ± 0.18	<0.001
	≥14 mm follicles		16.90 ± 7.33	14.12 ± 5.33	0.03
Mature follicles		15.36 ± 7.44	11.82 ± 5.40	0.008
Retrieved oocytes		14.52 ± 6.99	9.90 ± 5.68	<0.001
Oocytes M2		10.82 ± 5.79	6.96 ± 4.22	<0.001
Number of embryos		9.98 ± 5.49	6.78 ± 4.51	0.002
Ovulation cycles days		9.61 ± 1.03	10.36 ± 1.38	0.003
Endometrial thickness		8.07 ± 0.47	7.66 ± 1.57	0.08
Positive pregnancy**		15 (30%)	12 (24%)	0.49
Data presented as Mean ± SD or frequency (percent), * Independent samples t-test; ** Chi-square test				
LH: Luteinizing hormone; HCG: Human chorionic gonadotropin				

## 4. Discussion

In this study, we evaluated the effect of Utrogestan on preventing premature LH surge in IVF cycles and observed no LH surge in either the case or control groups.

Premature LH surge is a major problem in IVF cycles, reducing the efficacy of ovarian stimulation (9). Different therapies have been introduced to prevent or reduce LH surge, including GnRH agonists and antagonists (3-5). GnRH agonists are accompanied with a higher rate of ovarian hyperstimulation syndrome, whereas poor ovarian response and a lower rate of pregnancy are common with GnRH antagonist treatments (10). However, progesterone has been shown to properly reduce LH surge and is considered a substitute for previous therapies, particularly with its benefit of being taken orally rather than injected (11). Previous studies using medroxyprogesterone acetate reported no LH surge among normal responders (12, 13) or women with PCOS (14). Moreover, similar to our findings, Zhu and colleagues also reported no LH surge using Utrogestan (6, 7). One reason for this is that if administered during the follicular phase, progesterone can slow LH pulse frequency, thereby reducing LH levels (15). It has also been demonstrated that progesterone acts by affecting progesterone receptors in the hypothalamus, which suppresses LH surge (13, 16) and its effect is reversible. Progesterone can be administered via different routes such as oral, rectal, vaginal, and intramuscular (17), with all routes showing a similar efficacy regarding pregnancy, miscarriage, and live birth rates.

In this study, women treated with Utrogestan had significantly higher progesterone levels at the HCG injection day, more follicles of >14 mm with higher maturity, and more retrieved oocytes with a higher rate of embryos available for transfer. The pregnancy rate was also higher in the case group although not significant. Zhu and colleagues (18) studied the effects of Utrogestan in women with PCOS and observed a higher rate of fertilization, a higher rate of viable embryos per oocyte retrieved, and a higher pregnancy rate in the Utrogestan group than that in the control group. However, another study by Zhu and colleagues (6) that evaluated the effect of Utrogestan on IVF observed a similar number of oocytes retrieved with Utrogestan, hMG protocol, and short protocol, with no significant difference observed in the mature oocyte rate or clinical pregnancy rate. Studies comparing Utrogestan with other forms of progesterone have demonstrated no drug superiority in terms of achieving satisfactory results. In a study by Bergh and colleagues, no difference in the pregnancy rate between vaginal Utrogestan and crinone was found (19). Biberoglu and colleagues also observed no difference on comparing 300 mg and 600 mg vaginal Utrogestan (20).

The better outcome in the Utrogestan group in both this study and Zhu and colleagues' study in women with PCOS could be owing to the fact that as it is exogenous natural progesterone, it would induce an autoregulatory positive feedback action to enhance the production of endogenous progesterone, as indicated in previous animal studies (18, 21, 22). Various studies have indicated that progesterone affects the hypothalamic-pituitary-ovarian axis for premature LH surge suppression as well as blocks LH surge induced by estradiol (23). It has also been reported that administering progestin during the normal follicular phase could reduce LH levels in normal women (24). Thus, progesterone administration could suppress LH surge in IVF cycles. Compared with other treatments, Utrogestan can be orally administered, is well tolerated by users in terms of lower levels of stress and discomfort, and has economic benefits.

## 5. Conclusion

In conclusion, Utrogestan is an alternative treatment that could reduce the rate of premature LH surge and increase the reproductive outcome.

##  Conflicts of Interest

We have no conflicts of interest.

## References

[1] Hafiz P., Nematollahi M., Boostani R., Jahromi B. N. (2017). Predicting implantation outcome of in vitro fertilization and intracytoplasmic sperm injection using data mining techniques. *International Journal of Fertility & Sterility*.

[2] Hamdi K., Pia H., Hakimi P., Chaichi P. (2018). Relationship between thickness and pattern of endometrium and pregnancy rate in in vitro fertilization-intracytoplasmic. *Journal of Analytical Research in Clinical Medicine*.

[3] Stimpfel M., Vrtacnik-Bokal E., Pozlep B., Virant-Klun I. (2015). Comparison of GnRH agonist, GnRH antagonist, and GnRH antagonist mild protocol of controlled ovarian hyperstimulation in good prognosis patients. *International Journal of Endocrinology*.

[4] Dawood A. S., Algergawy A., Elhalwagy A. (2017). Reduction of the cetrorelix dose in a multiple-dose antagonist protocol and its impact on pregnancy rate and affordability: A randomized controlled multicenter study. *Clinical and Experimental Reproductive Medicine*.

[5] Rabati B. K., Zeidi S. N. (2012). Investigation of pregnancy outcome and ovarian hyper stimulation syndrome prevention in agonist and antagonist gonadotropin-releasing hormone protocol. *Journal of Research in Medical Sciences*.

[6] Zhu X., Zhang X., Fu Y. (2015). Utrogestan as an Effective Oral Alternative for Preventing Premature Luteinizing Hormone Surges in Women Undergoing Controlled Ovarian Hyperstimulation for In Vitro Fertilization. *Medicine*.

[7] Zhu Zhang XL., Fu YL. (2015). Effect of progesterone used to prevent LH surges in controlled ovarian stimulation. Reprod Contra. *Zhu XX*.

[8] Hutchens E. G., Ramsey K. A., Howard L. C., Abshire M. Y., Patrie J. T., McCartney C. R. (2016). Progesterone has rapid positive feedback actions on LH release but fails to reduce LH pulse frequency within 12 h in estradiol-pretreated women. *Physiological Reports*.

[9] Nayot D., Klachook S., Casper R. F. (2013). Nimodipine, a calcium channel blocker, delays the spontaneous LH surge in women with regular menstrual cycles: a prospective pilot study. *Reproductive Biology and Endocrinology*.

[10] Reichman D. E., Zakarin L., Chao K., Meyer L., Davis O. K., Rosenwaks Z. (2014). Diminished ovarian reserve is the predominant risk factor for gonadotropin-releasing hormone antagonist failure resulting in breakthrough luteinizing hormone surges in in vitro fertilization cycles. *Fertility and Sterility*.

[11] Messinis I. E., Vanakara P., Zavos A., Verikouki C., Georgoulias P., Dafopoulos K. (2010). Failure of the GnRH antagonist ganirelix to block the positive feedback effect of exogenous estrogen in normal women. *Fertility and Sterility*.

[12] Dong J., Wang Y., Chai W. R., Hong Q. Q., Wang N. L., Sun L. H., Long H., Wang L., Tian H., Lyu Q. F., Lu X. F., Chen Q. J., Kuang Y. P. (2017). The pregnancy outcome of progestin-primed ovarian stimulation using 4 versus 10 mg of medroxyprogesterone acetate per day in infertile women undergoing in vitro fertilisation: a randomised controlled trial. *BJOG: An International Journal of Obstetrics & Gynaecology*.

[13] Huang C., Chen G., Shieh M., Li H. (2018). An extremely patient-friendly and efficient stimulation protocol for assisted reproductive technology in normal and high responders. *Reproductive Biology and Endocrinology*.

[14] Wang Y., Chen Q., Wang N., Chen H., Lyu Q., Kuang Y. (2016). Controlled Ovarian Stimulation Using Medroxyprogesterone Acetate and hMG in Patients With Polycystic Ovary Syndrome Treated for IVF. *Medicine*.

[15] Clarke B. L., Khosla S. (2010). Female reproductive system and bone. *Archives of Biochemistry and Biophysics*.

[16] Denis-Robichaud J., LeBlanc S. J., Jones-Bitton A., Silper B. F., Cerri R. L. A. (2018). Pilot study to evaluate the association between the length of the luteal phase and estrous activity detected by automated activity monitoring in dairy cows. *Frontiers in Veterinary Science*.

[17] Child T., Leonard S. A., Evans J. S., Lass A. (2018). Systematic review of the clinical efficacy of vaginal progesterone for luteal phase support in assisted reproductive technology cycles. *Reproductive BioMedicine Online*.

[18] Zhu X., Ye H., Fu Y. (2016). The Utrogestan and hMG protocol in patients with polycystic ovarian syndrome undergoing controlled ovarian hyperstimulation during IVF/ICSI treatments. *Medicine*.

[19] Bergh C., Lindenberg S. (2012). A prospective randomized multicentre study comparing vaginal progesterone gel and vaginal micronized progesterone tablets for luteal support after in vitro fertilization/intracytoplasmic sperm injection. *Human Reproduction*.

[20] Biberoglu E. H., Tanrikulu F., Erdem M., Erdem A., Biberoglu K. O. (2016). Luteal phase support in intrauterine insemination cycles: A prospective randomized study of 300 mg versus 600 mg intravaginal progesterone tablet. *Gynecological Endocrinology*.

[21] Wei M., Mahady G. B., Liu D., Zheng Z. S., Lu Y. (2016). Astragalin, a flavonoid from Morus alba (mulberry) increases endogenous estrogen and progesterone by inhibiting ovarian granulosa cell apoptosis in an aged rat model of menopause. *Molecules*.

[22] Peluso J. J., Pru J. K. (2014). Non-canonical progesterone signaling in granulosa cell function. *Reproduction*.

[23] Wang Y., Chen Q., Wang N., Chen H., Lyu Q., Kuang Y. (2016). Controlled Ovarian Stimulation Using Medroxyprogesterone Acetate and hMG in Patients With Polycystic Ovary Syndrome Treated for IVF. *Medicine*.

[24] Kim S. H., Burt Solorzano C. M., McCartney C. R. (2018). Progesterone administration does not acutely alter LH pulse secretion in the mid-follicular phase in women. *Physiological Reports*.

